# Graphite Felt Modified by Atomic Layer Deposition with TiO_2_ Nanocoating Exhibits Super-Hydrophilicity, Low Charge-Transform Resistance, and High Electrochemical Activity

**DOI:** 10.3390/nano10091710

**Published:** 2020-08-29

**Authors:** Wen-Jen Lee, Yu-Ting Wu, Yi-Wei Liao, Yen-Ting Liu

**Affiliations:** 1 Department of Applied Physics, National Pingtung University, Pingtung 90003, Taiwan; sagessetim@gmail.com; 2 Department of Applied Chemistry, National Pingtung University, Pingtung 90003, Taiwan; aleweichart@gmail.com (Y.-W.L.); vancleefezz@gmail.com (Y.-T.L.)

**Keywords:** graphite, graphite felt, carbon felt, atomic layer deposition, titanium dioxide, surface modification, hydrophilicity, electrochemical activity

## Abstract

Graphite felt (GF) is a multi-functional material and is widely used as electrodes of electrochemical devices for energy and environmental applications. However, due to the inherent hydrophobicity of graphite felt, it must be hydrophilically pretreated to obtain good electrochemical activity. Metal oxides coating is one of the feasible methods to modify the surface of GF, and in order to ensure that the metal oxides have a better conductivity for obtaining higher electrochemical activity, a subsequent H_2_ heat-treatment process is usually adopted. In this study, atomic layer deposition (ALD) is used to deposit TiO_2_ nanocoating on graphite felt (GF) for surface modification without any H_2_ thermal post-treatment. The results show that the ALD-TiO_2_-modified GF (ALD-TiO_2_/GF) owns excellent hydrophilicity. Moreover, the ALD-TiO_2_/GF exhibits excellent electrochemical properties of low equivalent series resistance (R_s_), low charge-transfer resistance (R_ct_), and high electrochemical activity. It demonstrates that ALD is an applicable technique for modifying the GF surface. In addition, it can be reasonably imagined that not only TiO_2_ film can effectively modify the GF surface, but also other metal oxides grown by ALD with nanoscale-thickness can also obtain the same benefits. We anticipate this work to be a starting point for modifying GF surface by using ALD with metal oxides nanocoating.

## 1. Introduction

Graphite felt (GF) has a three-dimensional (3D) porous structure with high specific surface area [1,2] and numerous outstanding properties such as excellent electrical conductivity, flexibility, corrosion resistance and electrochemical stability [3,4]. Therefore, it has been widely studied as electrodes for energy and environmental applications that include vanadium redox flow batteries (VRFBs) [5,6], super capacitors [7,8,9,10], microbial fuel cells (MFCs) [11,12], biofuel cells (BFCs) [13,14], electro-Fenton (EF) process [15,16,17] and so forth.

However, GF has a hydrophobic surface property in nature, resulting in its poor electrochemical activity in aqueous solution and a low performance of GF-based electrochemical devices [18]. In order to improve the electrochemical activity of GF, the hydrophobic surface of original GF should be modified to hydrophilic surface, moreover, the GF should be maintained native good electrical conductivity simultaneously.

Kozbial et al. reported [19] that the hydrophobicity of graphite surface is attributed to the adsorption of hydrophobic organic pollutants (i.e., hydrocarbons) in the atmospheric environment and the graphite surface can be hydrophilic if the adsorbed organic pollutants are removed. In addition, Le et al. indicated that attaching oxygen-containing functional groups to the surface of GF can effectively increase its hydrophilicity and electrochemical activity [18].

Several methods have been successfully applied to modify the GF surface at various conditions, including plasma treatment [20,21], chemical treatment [22,23,24,25,26,27], thermal treatment [28,29,30,31,32], nitrogenization treatment [33,34,35], carbon nanomaterials based modification [36,37,38,39], nanostructural metals [40,41,42,43] and metal oxides [44,45] decorating.

In general, an appropriate method for surface-modification of GF must include the following requirements. Firstly, the method should change the GF surface from hydrophobic to hydrophilic property to obtain a low interface resistance (low charge-transfer resistance, R_ct_) between the modified GF-based electrode and electrolyte for fast charge-transfer reactions. Secondly, the modified GF should maintain primitive excellent conductivity of original GF to receive a low equivalent series resistance (R_s_). To improve the electrochemical activity of GF, all of the above characteristics should be satisfied simultaneously. Finally, the modified GF must maintain long-term stability of hydrophilicity and excellent chemical stability to sustain corrosive electrolyte of electrochemical devices such as vanadium redox flow batteries (VRFBs), aluminum-ion batteries and so forth.

The GF modified with metal oxides decorating or coating exhibits long-term stable hydrophilicity and chemical stability. However, the conductivity of metal oxides is generally varied with the impurity and defect concentration in the oxides. If the metal oxides have poor conductivity, the conductivity of GF will decrease and result in its poor electrochemical activity. Therefore, in order to enhance the electrochemical activity of GF modified with metal oxides, typically a post-annealing process in a reducing atmosphere (containing H_2_ gas) is necessary. Because the concentration of oxygen vacancies in the lattice of metal oxides will be increased during the H_2_ thermal treatment process to form defect-rich non-stoichiometric metal oxides, a lower resistance of metal oxides for obtaining higher electrochemical activity is found [46,47].

In this work, a nanocoating TiO_2_ film (about 10 nm) is grown on the GF surface by atomic layer deposition (ALD) for surface-modification of GF and without any H_2_ thermal treatment process. TiO_2_ is a native hydrophilic material with outstanding chemical stability. Thus, the hydrophobic surface of GF can be modified to hydrophilic by coating with TiO_2_ films, and the TiO_2_ surface-coating can withstand the corrosion of strong corrosive electrolyte when the TiO_2_-modified GF is applied to electrochemical devices. ALD is an advanced technique for growing highly conformal thin films on the surfaces of three-dimensional complex-structures and nanostructures with atomic-level thickness control and excellent coating uniformity [48,49,50]. Moreover, conformal TiO_2_ thin films with nanoscale-thickness have been successfully coated on carbon nanotube (CNT) fibers and graphite fibers by using ALD technique [51,52,53]. Therefore, a 10-nm-thick TiO_2_ film can be uniformly coated on the GF surface by ALD technology with accurate control of film thickness at the nanoscale. We make the TiO_2_ film with an ultra-thin thickness of 10 nm and a nanocrystalline structure, which not only confers hydrophilicity to GF but also ensures that the ALD-TiO_2_-modified GF (ALD-TiO_2_/GF) has a sufficiently low resistance for achieving excellent electrochemical activity (the reason and design concept will be explained later).

The results show that the ALD-TiO_2_/GF has a super-hydrophilic property and retains a good conductivity, the same as the original GF (see Supplementary Video and Materials, Figure S1). The electrochemical measurements of cyclic voltammetry (CV) and electrochemical impedance spectroscopy (EIS) show that the electrochemical activity of GF is significantly improved by an order of magnitude of two via ALD-TiO_2_ modification. Besides, both original GF and ALD-TiO_2_/GF have a similar low equivalent series resistance (R_s_), which is about 5.85 and 3.62 Ω for original GF and ALD-TiO_2_/GF, respectively. However, ALD-TiO_2_/GF has a charge-transfer resistance (R_ct_) of about 0.28 Ω, which is 100 times lower than the original GF of about 24.88 Ω.

As the excellent electrochemical properties, it is expected that the ALD-TiO_2_/GF electrode has potential applications in vanadium redox flow batteries (VRFB), aluminum-ion batteries, and other electrochemical energy-storage devices. Additionally, this work demonstrates that ALD technology is an applicable method for surface modification of GF. It is believed that not only TiO_2_ films are effective in modifying GF surface but also other metal oxide films grown by ALD with nanoscale thickness can be applied for surface modification of GF.

## 2. Materials and Methods

### 2.1. Surface Modification of GF

For surface modification of GF, a low-pressure ALD system was used to coat a thin TiO_2_ film on the surface of GF (the thickness of GF is about 2 mm). The process includes 3 steps, as follows:

(1) Removing the surface-adsorbed organic pollutants of GF: the original GF was introduced into the ALD reactor and then the GF was annealed at 500 °C for 2 h in the ALD reactor. The purpose of this step is to remove organic pollutants adsorbed on the surface of GF by thermal decomposition, to ensure that the GF has a clean and hydrophilic surface for ALD process.
(2) Ultra-thin amorphous TiO_2_ film coated on the GF surface by ALD: a 10-nm-thick TiO_2_ film was coated on the GF surface by ALD with 100 ALD-cycles at 60 °C for which the details of the ALD process were described in our previous works [54,55,56]. Briefly, TiCl_4_ and H_2_O were used as the precursors, Ar was used as the purge gas, and the growth rate is about 0.1 nm per cycle. In this step, an ultra-thin amorphous TiO_2_ film is uniformly coated on all the surfaces of the GF to form ALD-TiO_2_/GF sample.
(3) Nanocrystallization of TiO_2_ film: TiO_2_ grown at the low temperature of 60 °C has an amorphous structure. In order to improve the corrosion resistance and activity of TiO_2_, a post-annealing process was performed at 500 °C for 2h in the ALD reactor to transform the TiO_2_ surface coating from an amorphous into an anatase crystal structure.

#### Design Concept of This Study

**Figure 1:**
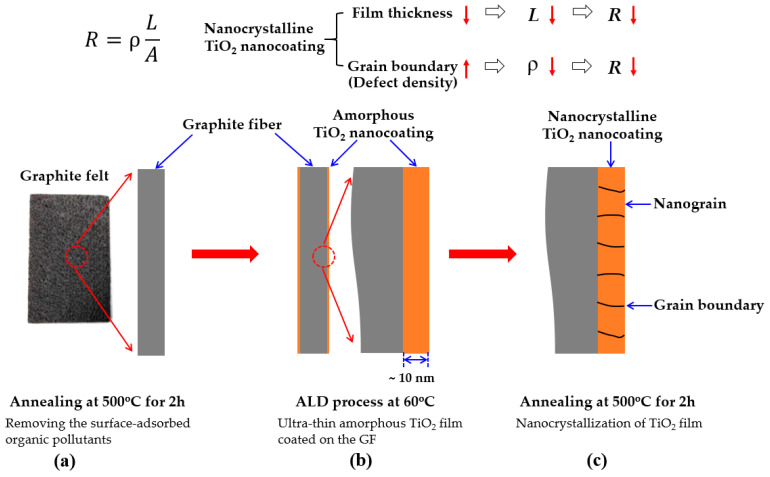
Schematic diagram of the design concept of this study. (**a**) Removing the surface-adsorbed organic pollutants of GF by thermal annealing process, (**b**) Ultra-thin amorphous TiO_2_ film coated on the GF surface by ALD, and (**c**) Nanocrystallization of TiO_2_ film by thermal annealing process.

The design concept of the above process is shown in Figure 1. The well-known Equation (1) shows the relationship between the resistance, resistivity, length, and area of a material.

(1) $$R=\rho \frac{L}{A}$$

where *R* is the resistance of the material, ρ is the resistivity of the material, *L* is the transmission length of charges transfer in the material, and *A* is the cross-sectional area of material perpendicular to the current direction. According to the Equation (1), it can be known that if we reduce ρ, shorten *L* or increase *A*, then the *R* will be reduced.

In this work, ρ is the resistivity of TiO_2_ film, *L* is the thickness of TiO_2_ film, and *A* is the surface area of graphite felt for TiO_2_ film deposition. Thus, reducing ρ and *L* of TiO_2_ film are the developing directions that can be striven in this work. In this study, in order to reduce the resistance (*R*) of the TiO_2_ coating on the GF surface, on the one hand, a nanoscale-thick (~10 nm) TiO_2_ film was grown on the GF to shorten *L*. On the other hand, a two-step process (firstly growing an amorphous film at low temperature, and then raising the temperature to crystallize the film) was used to obtain a nanocrystalline TiO_2_ film. It is noticed that my previous work [55] and other previous studies [51,52] have confirmed that nanocrystalline TiO_2_ films can be obtained by the two-step process. Since the nanocrystalline TiO_2_ film has a large amount of grain boundary defects, the resistivity (ρ) of the TiO_2_ film can be effectively reduced. As described above, the TiO_2_ film with an ultra-thin thickness of 10 nm and a nanocrystalline structure can succeed a low resistance.

### 2.2. Characterizations

Scanning electron microscopy (SEM) images and energy-dispersive X-ray spectroscopy (EDS) mapping analyses were carried out on a high-resolution scanning electron microscope (SU8000, Hitachi, Japan) provided with an energy-dispersive X-ray spectroscope (XFlash 5060FQ, Bruker, Germany). X-ray diffraction (XRD) patterns were performed on an X-ray diffractometer (D8 Advance Eco, Bruker, Germany). Raman spectra of the samples were examined by a micro Raman spectrometer (UniRAM II, Uninanotech, Korea).

### 2.3. Electrochemical Analysis

**Figure 2:**
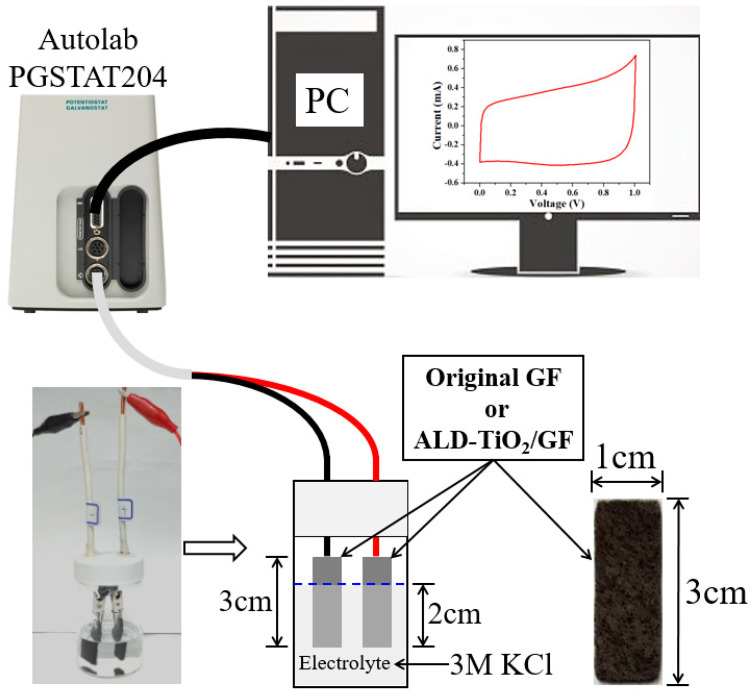
Schematic diagram of the electrochemical measurements.

Figure 2 displays a schematic diagram of the electrochemical measurements. In order to enhance the difference between original GF and ALD-TiO_2_-modified GF (ALD-TiO_2_/GF), two pieces of original GFs or ALD-TiO_2_/GFs with a length of 3 cm and a width of 1cm were used as electrodes and a 3M KCl aqueous solution was used as electrolyte, to configure two-electrode cells for electrochemical analyses. The electrochemical measurements of cyclic voltammetry (CV) and electrochemical impedance spectroscopy (EIS) were examined in the two-electrode cells by using a potentiostat (Autolab PGSTAT204, Metrohm AG, Switzerland). In addition, an electrochemistry software (Nova, Metrohm AG, Switzerland) was used to analyze the data of electrochemical measurements.

## 3. Results and Discussion

**Figure 3:**
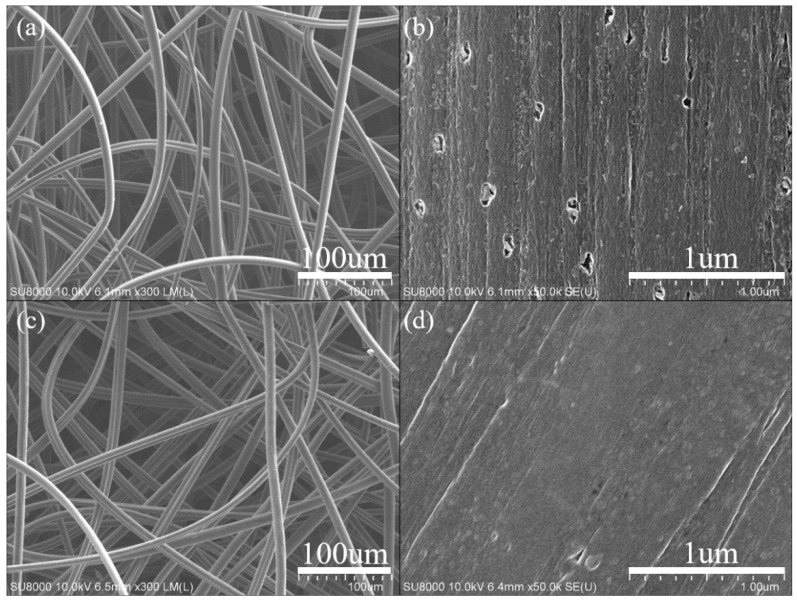
SEM images of original graphite felt (**a**,**b**) and atomic layer deposition (ALD)-TiO_2_-modified graphite felt (**c**,**d**).

**Figure 4:**
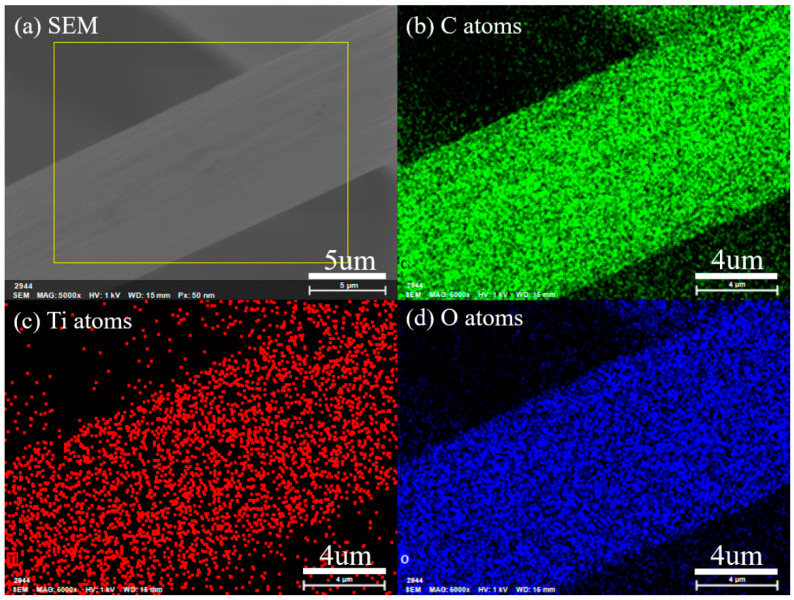
Energy-dispersive X-ray spectroscopy (EDS) mapping analysis of ALD-TiO_2_-modified graphite felt. (**a**) SEM image of the selected-area EDS, and the elemental distribution images of (**b**) C, (**c**) Ti and (**d**) O atoms.

The SEM images of original GF and ALD-TiO_2_-modified GF (ALD-TiO_2_/GF) are shown in the Figure 3. It is clearly showed that the GF is constructed from numerous carbon fibers with a diameter in the range of about 7–12 µm. Moreover, both original GF and ALD-TiO_2_/GF have almost the same micromorphology (compare Figure 3a with Figure 3c) because the surface-coating of TiO_2_ film is only 10 nm. However, the high-resolution images (Figure 3b,d) show that ALD-TiO_2_/GF has a smoother surface than original GF and the surface nanopores of original GF are filled by ALD-TiO_2_ coating. Figure 4 shows the elemental mapping images of selected-area EDS analysis for a carbon fiber of ALD-TiO_2_/GF. It reveals that Ti and O atoms are distributed homogeneously on the carbon fiber, demonstrating that the thin TiO_2_ film is coated uniformly on the GF by ALD.

**Figure 5:**
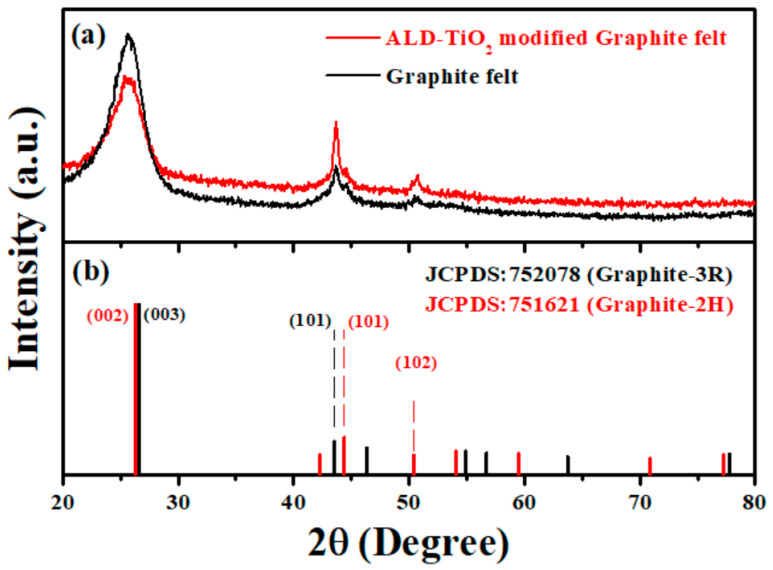
(**a**) XRD patterns of original graphite felt and ALD-TiO_2_ modified graphite felt, (**b**) JPCDS cards of Graphite-3R and Graphite-2H.

**Figure 6:**
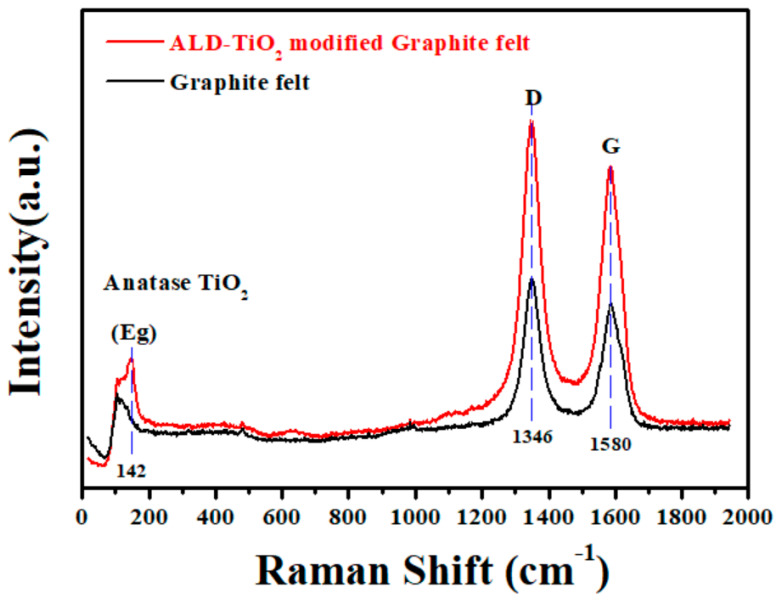
Raman spectra of original graphite felt and ALD-TiO_2_-modified graphite felt.

Figure 5 shows the XRD patterns of original graphite felt (GF) and ALD-TiO_2_-modified graphite felt (ALD-TiO_2_/GF). Obviously, XRD peaks located at 2θ of 25.6, 43.6, 44.5 and 50.4° are observed in the XRD patterns that the XRD peaks of 25.6, 44.5 and 50.4° are corresponded to the (002), (101) and (102) planes of hexagonal Graphite-2H, and the XRD peaks of 25.6 and 43.6° can be indexed to the (003) and (101) planes of rhombohedral Graphite-3R, demonstrating that the crystal structure of GF is constructed from Graphite-2H with Graphite-3R. In addition, the XRD pattern of ALD-TiO_2_/GF is almost the same as original GF and no XRD peak of TiO_2_ crystal is detected because the thickness of TiO_2_ surface coating is very thin (only about 10 nm), which is difficult to detect by XRD. Therefore, the samples are further analyzed by Raman spectrometer and the results are shown in the Figure 6.

In the Figure 6, two strong Raman peaks located at wavenumber 1346 and 1580 cm^−1^ are detected in the both original GF and ALD-TiO_2_/GF. The Raman peaks of 1346 and 1580 cm^−1^ are indexed to the D-band (sp^3^ carbon networks) and G-band (sp^2^ carbon networks) of graphite, respectively [57,58]. In addition, a weak Raman peak located at around 142 cm^−1^ is also observed in the Raman spectrum of the ALD-TiO_2_/GF, which can be indexed to the Eg mode of anatase TiO_2_ [59,60,61,62], demonstrating that a thin anatase TiO_2_ layer is coated on the GF surface.

**Figure 7:**
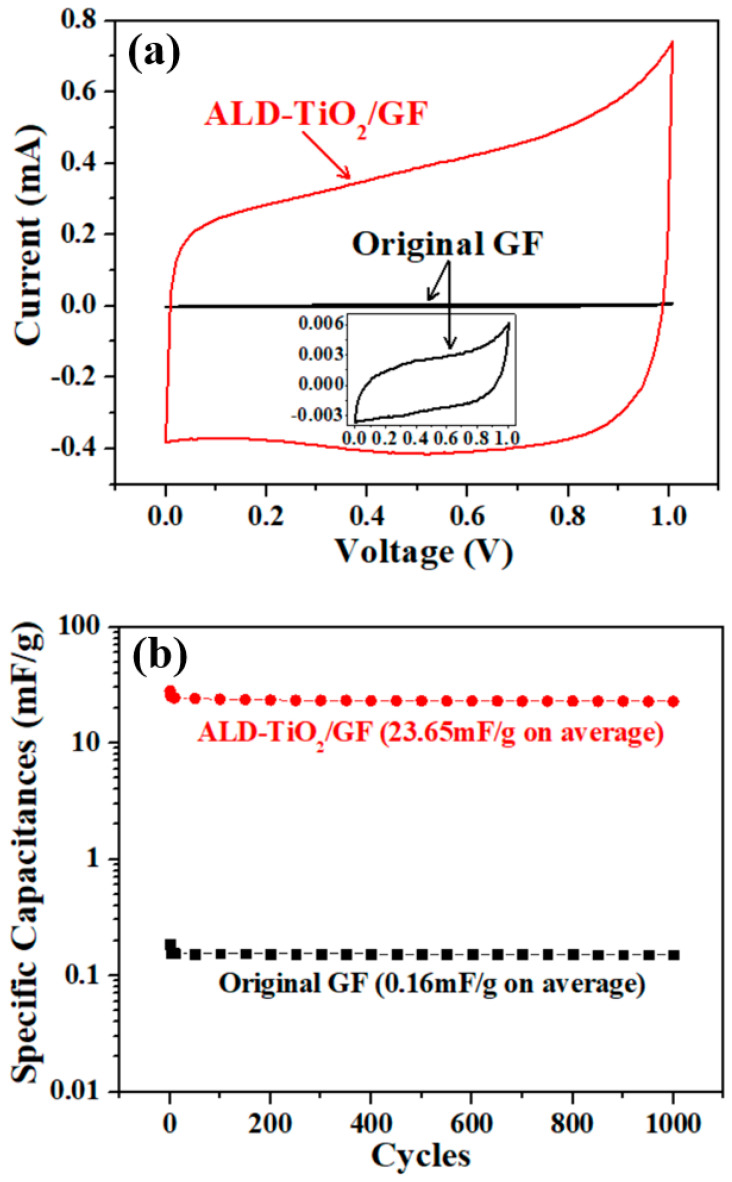
(**a**) Cyclic voltammetry (CV) curves of the original graphite felt (GF) and ALD-TiO_2_ modified GF (ALD-TiO_2_/GF), the scan rate is 1 V/s. (**b**) Specific capacitances of original GF and ALD-TiO_2_/GF obtained from 1000 times CV cycling-test.

In order to evaluate the electrochemical properties of original GF and ALD-TiO_2_/GF, electrochemical measurements of cyclic voltammetry (CV) and electrochemical impedance spectroscopy (EIS) were performed. Figure 7a shows the CV curves of original GF and ALD-TiO_2_/GF selected from the 10th cycle. Besides, the specific capacitances (calculated from CV measurements) of these electrodes as a function of cycle index are shown in Figure 7b. As shown in Figure 7a, the CV curve of ALD-TiO_2_/GF exhibits quasi-rectangular shape and the area of CV curve of ALD-TiO_2_/GF is significantly larger than the original GF. As shown in Figure 7b, the specific capacitances are about 0.16 and 23.65 mF/g on average for original GF and ALD-TiO_2_/GF, respectively. It can be clearly seen that the electrochemical activity of GF is improved an order of magnitude of two. Besides, the ALD-TiO_2_/GF has also demonstrated long-term stability, which is verified by a CV-test of 1000 cycles. The experimental data of 1000 times CV cycling-test are shown in the Supplementary Materials (Figure S2).

The enhancement of electrochemical activity of GF by ALD-TiO_2_ surface modification can be reasonably attributed to the hydrophobic surface of GF which has been changed to super-hydrophilic, therefore, the equivalent series resistance (R_s_) and charge-transfer resistance (R_ct_) of between ALD-TiO_2_/GF electrodes and electrolyte can be effectively reduced. In order to prove that the R_s_ and R_ct_ of ALD-TiO_2_/GF electrodes are lower than original GF, the measurements of electrochemical impedance spectroscopy (EIS) were carried out. The EIS is a powerful tool, which has been widely used for analyzing the internal resistance of electrodes and the resistance between the electrode and electrolyte [63,64,65].

**Figure 8:**
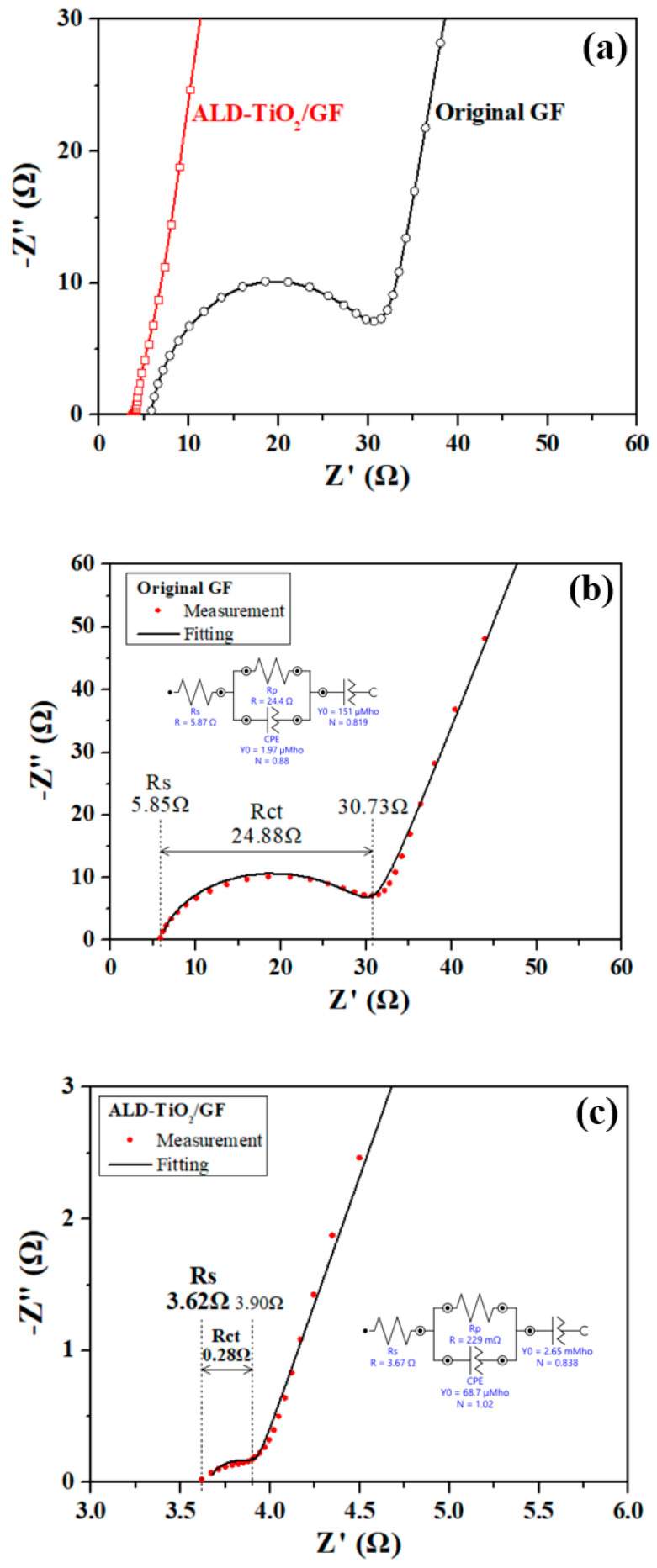
Nyquist plots of the original GF and ALD-TiO_2_/GF measured by electrochemical impedance spectroscopy (EIS) measurement. (**a**) Compared figure of the original GF and ALD-TiO_2_/GF, (**b**) and (**c**) show the experimental (red dots) and fitting (black line) data for the original GF and ALD-TiO_2_/GF, respectively.

Figure 8 reveals the Nyquist plots of the original GF and ALD-TiO_2_/GF performed by EIS measurement. The Figure 8 exhibits a clear evidence that the equivalent series resistance (R_s_) and charge-transfer resistance (R_ct_) of ALD-TiO_2_/GF electrodes are lower than the original GF. The R_s_ and R_ct_ are about 5.85 and 24.88 Ω for the original GF (as Figure 8b) and are about 3.62 and 0.28 Ω for ALD-TiO_2_/GF (as Figure 8c). Besides, it is noticed that both the original GF and ALD-TiO_2_/GF have a similar R_s_ value. However, the R_ct_ value of ALD-TiO_2_/GF is 100 times lower than that of the original GF, proving that the significant increase (100 times) in electrochemical activity is primarily due to the contribution of the decrease in the R_ct_ value between the electrode and the electrolyte.

## 4. Conclusions

In conclusion, ALD technique with TiO_2_ nanocoating is successfully employed as a new method for surface modification of GF to obtain excellent hydrophilicity, low equivalent series resistance (R_s_), low charge-transfer resistance (R_ct_), and high electrochemical activity. Especially, no any H_2_ thermal post-treatment is required. These excellent properties make ALD-TiO_2_/GF have the potential to be used in electrochemical energy storage devices. We are currently conducting research and process optimization on applying ALD-TiO_2_/GF to vanadium redox flow batteries (VRFBs). In addition, it can be reasonably anticipated that not only TiO_2_ but also other metal oxides with nanoscale-thickness grown by ALD can be possibly applied to effectively modify the GF surface.

## Supplementary Materials

A supplementary video and Supplementary Materials are available online at https://www.mdpi.com/2079-4991/10/9/1710/s1, The video demonstrates a simple-test of conductivity and hydrophilicity of original GF and ALD-TiO_2_/GF. Figure S1: Simple-test for conductivity and hydrophilicity of original GF and ALD-TiO_2_/GF. Figure S2: 1000 times CV cycling-test of original GF and ALD-TiO_2_/GF.
