# Supplementary material for: Graphite Felt Modified by Atomic Layer Deposition with TiO2 Nanocoating Exhibits Super-Hydrophilicity, Low Charge-Transform Resistance, and High Electrochemical Activity
Source: Nanomaterials (Basel). 2020 Aug 29;10(9):1710. doi: 10.3390/nano10091710 (PMC7560090; doi:10.3390/nano10091710)
Supplement: Supplementary file 1 [file nanomaterials-10-01710-s001.zip › Approved Manuscript/Supplementary Materials.docx]

**Supplementary materials**

Graphite Felt Modified by Atomic Layer Deposition with TiO_2_ Nanocoating Exhibits Super-Hydrophilicity, Low Charge-Transform Resistance, and High Electrochemical Activity

Wen-Jen Lee ^1,^*, Yu-Ting Wu ^1^, Yi-Wei Liao ^2^ and Yen-Ting Liu ^2^

^1^ Department of Applied Physics, National Pingtung University, Pingtung 90003, Taiwan; [sagessetim@gmail.com](mailto:sagessetim@gmail.com)

^2^ Department of Applied Chemistry, National Pingtung University, Pingtung 90003, Taiwan; [aleweichart@gmail.com](mailto:aleweichart@gmail.com) (Y.-W.L.); [vancleefezz@gmail.com](mailto:vancleefezz@gmail.com) (Y.-T.L.)

***** Correspondence: wenjenlee@mail.nptu.edu.tw; Tel.: +886-8-7663800

**Table of Contents**

1. **Supplementary Video Legends** ……………………….………….………………. **Page 2.**
2. **Figure S1.** **Simple test for conductivity and hydrophilicity** ……………………. **Page 3.**
3. **Figure S2.** **1000 times CV cycling-test** …………………………………………… **Page 4.**

**1) Supplementary Video Legends**

File name: Supplementary Video.wmv

Full length: 1 min 2 sec.

**Video contents:**

1. The 1^st^ part (time from 00:00:00 to 00:00:10): Using a simple multimeter to test the conductivity of the original GF and ALD-TiO_2_/GF, it shows that both the original GF and ALD-TiO_2_/GF have almost the same good conductivity (resistance less than 5Ω).

2. The 2^nd^ part (time from 00:00:11 to 00:00:44): Using water droplets to test the hydrophilicity of the original GF and ALD-TiO_2_/GF. The result shows that the original GF is super-hydrophobic (the water droplets are spherical), while ALD-TiO_2_/GF is super-hydrophilic (the water droplets penetrate directly into the GF).

3. The 3^rd^ part (time from 00:00:45 to 00:01:02): Throw the original GF and ALD-TiO_2_/GF into the water, it shows that the original GF cannot sink into water because of its super-hydrophobicity, while ALD-TiO_2_/GF has super-hydrophilicity and sinks directly into water quickly。

**2) Figure S1. Simple test for conductivity and hydrophilicity of original GF and ALD-TiO_2_/GF**


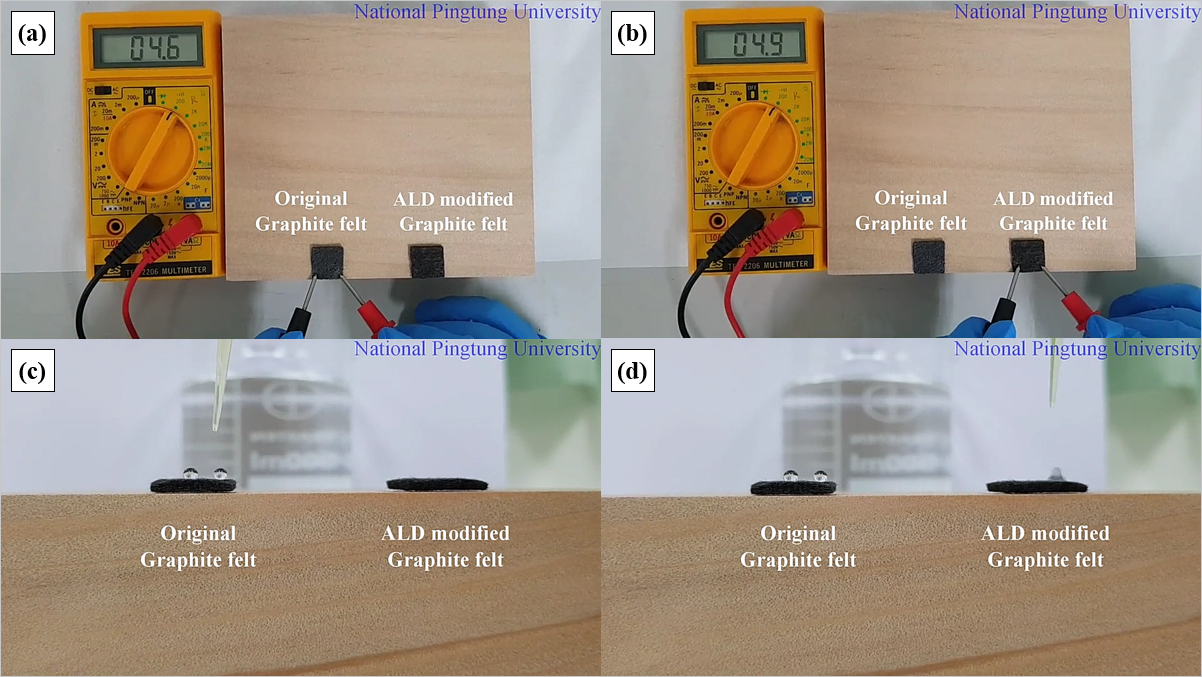


**
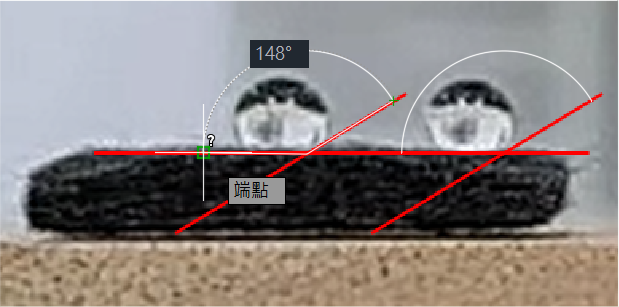

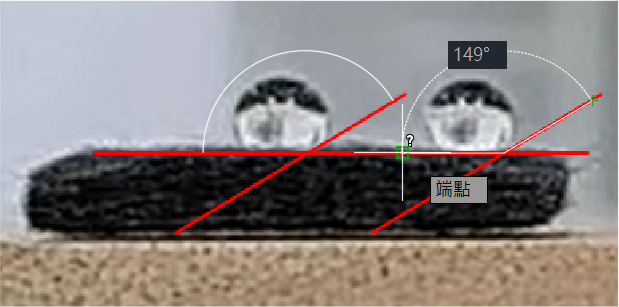
**

**Figure S1.** This set of pictures was taken from the Supplementary Video. The (a) and (b) show the measurement of resistance by using a simple multimeter for the original GF and ALD-TiO_2_/GF. The (c) and (d) show a simple test for the hydrophilicity of the original GF and ALD-TiO_2_/GF. The results show that both the original GF and ALD-TiO_2_/GF have almost the same good conductivity (resistance less than 5Ω), the original GF has a super- hydrophobic surface and the ALD-TiO_2_/GF has a super-hydrophilic surface. In addition, the bottom pictures show the contact angle of the original GF.

**3) Figure S2. 1000 times CV cycling-test of original GF and ALD-TiO_2_/GF**


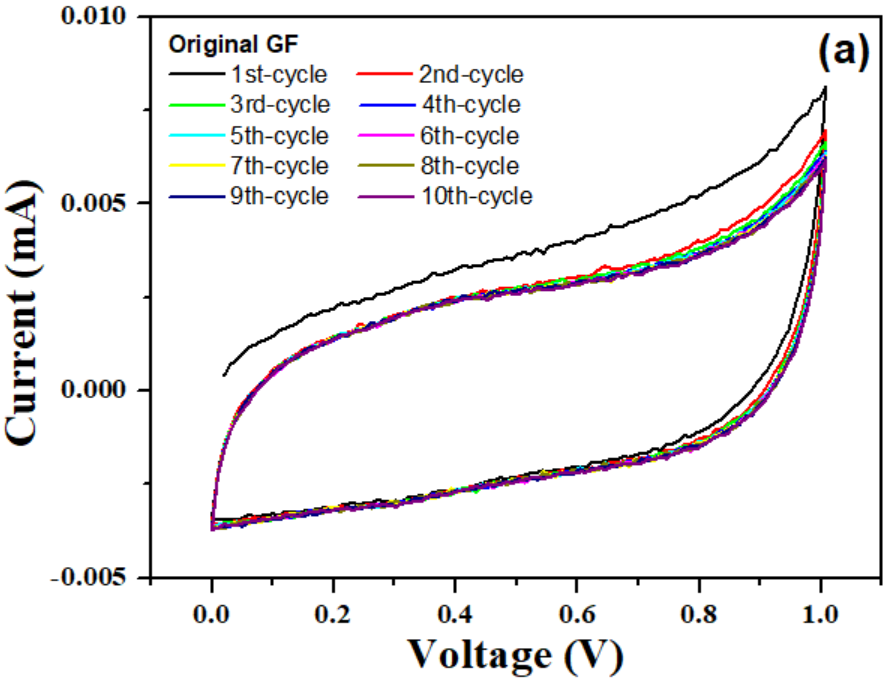

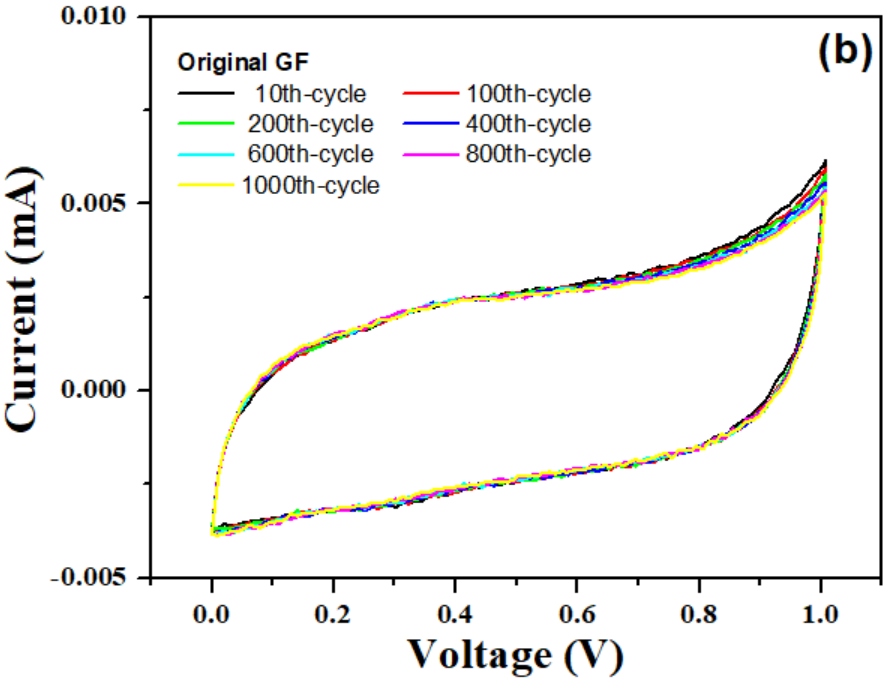


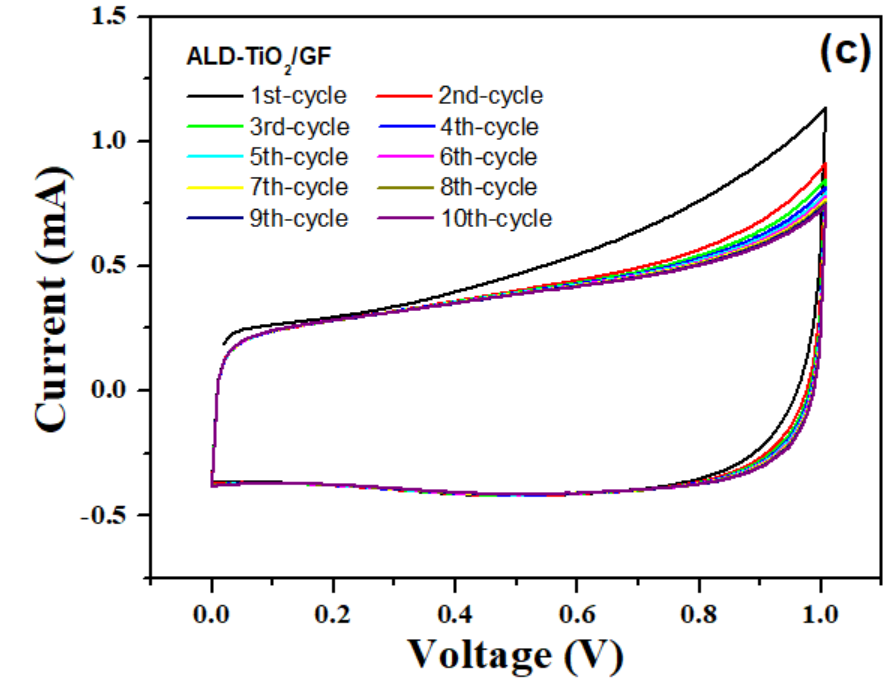

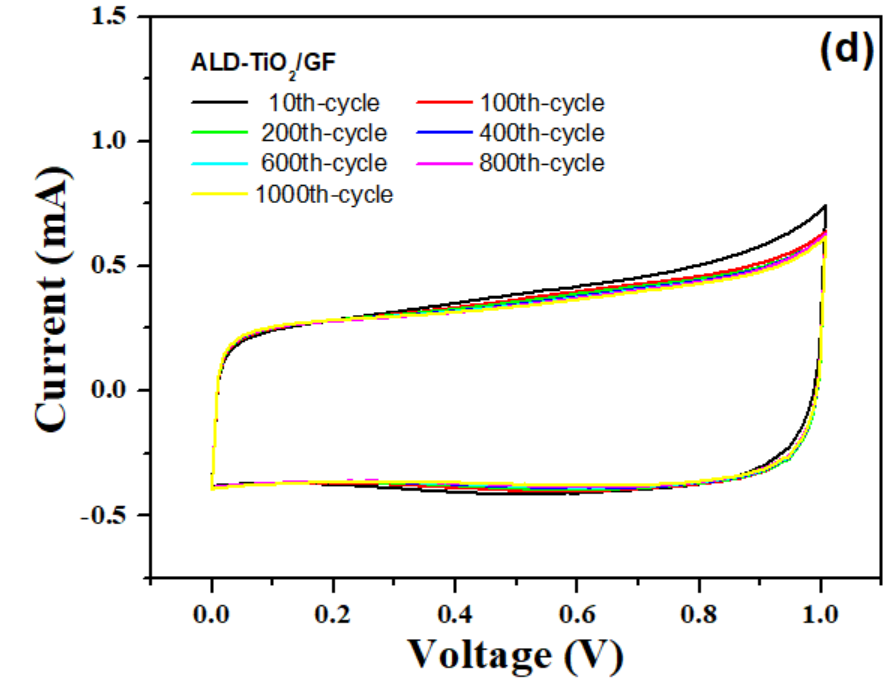


Figure S2. 1000 times CV cycling-test of original GF (a, b) and ALD-TiO_2_/GF (c, d).
